# Molecular characterization of *tlyA *gene product, Rv1694 of *Mycobacterium tuberculosis*: A non-conventional hemolysin and a ribosomal RNA methyl transferase

**DOI:** 10.1186/1471-2091-11-35

**Published:** 2010-09-20

**Authors:** Aejazur Rahman, Saumya S Srivastava, Amita Sneh, Neesar Ahmed, Musti V Krishnasastry

**Affiliations:** 1 National Centre for Cell Science, Ganeshkhind Road, Pune - 411007, Maharashtra, India

## Abstract

**Background:**

*Mycobacterium tuberculosis *is a virulent bacillus causing tuberculosis, a disease responsible for million deaths each year worldwide. In order to understand its mechanism of pathogenesis in humans and to help control tuberculosis, functions of numerous *Mycobacterium tuberculosis *genes are being characterized. In this study we report the dual functionality of *tlyA *gene product of *Mycobacterium tuberculosis *annotated as Rv1694, a 268 amino acid long basic protein.

**Results:**

The recombinant purified Rv1694 protein was found to exhibit hemolytic activity *in vitro*. It showed concentration and time-dependent hemolysis of rabbit and human erythrocytes. Multiple oligomeric forms (dimers to heptamers) of this protein were seen on the membranes of the lysed erythrocytes. Like the oligomers of conventional, well-known, pore-forming toxins, the oligomers of Rv1694 were found to be resistant to heat and SDS, but were susceptible to reducing agents like β-mercaptoethanol as it had abolished the hemolytic activity of Rv1694 indicating the role of disulfide bond(s). The Rv1694 generated *de novo *by *in vitro *transcription and translation also exhibited unambiguous hemolysis confirming the self assembly and oligomerization properties of this protein. Limited proteolytic digestion of this protein has revealed that the amino terminus is susceptible while in solution but is protected in presence of membrane. Striking feature of Rv1694 is its presence on the cell wall of *E. coli *as visualized by confocal microscopy. The surface expression is consistent with the contact dependent haemolytic ability of *E. coli *expressing this protein. Also, immune serum specific to this protein inhibits the contact dependent hemolysis. Moreover, Rv1694 protein binds to and forms stable oligomers on the macrophage phagosomal membranes. In addition to all these properties, *E. coli *expressing Rv1694 was found to be susceptible to the antibiotic capreomycin as its growth was significantly slower than mock vector transformed *E. coli*. The S30 extract of *E. coli *expressing the Rv1694 had poor translational activity in presence of capreomycin, further confirming its methylation activity. Finally, incorporation of methyl group of [^3^H]-S-adenosylmethionine in isolated ribosomes also confirmed its methylation activity.

**Conclusions:**

The Rv1694 has an unusual dual activity. It appears to contain two diverse functions such as haemolytic activity and ribosomal RNA methylation activity. It is possible that the haemolytic activity might be relevant to intra-cellular compartments such as phagosomes rather than cell lysis of erythrocytes and the self-assembly trait may have a potential role after successful entry into macrophages by *Mycobacterium tuberculosis*.

## Background

The complete genome sequence of the *Mycobacterium tuberculosis (M. tb)*, H37Rv, has been determined and its bioinformatic analysis improved our understanding of the life-cycle of this facultative, intracellular, human pathogen [1]. Although re-annotation of the genome sequence of *M. tb *strain, H37Rv, changed some assignments, the significant portion of the annotation remained unchanged [2].

Bioinformatic analysis has allowed us to predict the function for 2058 proteins out of 3995 (52%) and more than 150 of these have been experimentally identified in mycobacterial function. Among the 1051 conserved hypothetical proteins, 376 putative proteins showed no similarity to any known protein and it is possible that some of them may be specific to *M. tb *only [3-7]. In H37Rv, among many virulence factors, it was predicted that *tlyA *gene is a part of an operon containing at least three other genes viz. the first being *tlyA *(Rv1694), second is *ppnk *(Rv1695) and the third is RecN (Rv1696), homologous to *E. coli *RecN [8]. Since H37Rv is a well characterized pathogen, an obvious question surfaces about the role of Rv1694, with respect to its pathogenicity and virulence because during the initial annotation, Rv1694 was predicted to be a 268 amino acid long, hemolytic protein, which may function as a virulence determinant as it showed homology with the *tlyA *gene products of swine pathogen *Serpulina hyodysenteriae*, a causative agent of swine dysentery and almost identical to *tlyA *of *Mycobacterium leprae *[9,10]. This annotation was consistent with experimental observations that described the presence of hemolytic molecules in *M. tb*. For example, King and co-workers detected contact dependent hemolytic activity in the virulent strain H37Rv which was reduced by several fold in attenuated strain H37Ra, and not present in vaccine strain BCG [11]. Moreover, two more proteins of *M. tb*, which showed homology with Phospholipase C of *Pseudomonas aeruginosa *and 30% homology to hemolysin A, precursor of *Vibreo cholerae*, which have been reported to possess hemolytic activity [12-14]. In addition, Wren *et al*. showed the presence of *tlyA *homologues in *M. tb*, *Mycobacterium leprae*, *Mycobacterium avium *and *Mycobacterium bovis *BCG, but appeared to be absent in non-pathogenic strain *Mycobacterium smegmatis*. Interestingly, introduction of the *tlyA *gene into *Mycobacterium smegmatis *using a mycobacterial shuttle expression plasmid increased the contact dependent hemolysis orchestrated by it [8]. However, no further information is available on Rv1694 until recent studies showed that Rv1694 functions as a ribosomal RNA methyltransferase [15]. The methylase activity resulted in methylation of ribosomal RNA which can alter the susceptibility to capreomycin, a macrocyclic peptide antibiotic. It has been observed that mutations in the *tlyA *gene may make the bacteria resistant towards capreomycin [15,16]. Hence, the question arises as to whether the *tlyA *is a hemolysin or a ribosomal methyltransferase or it is a dually active molecule.

Multi-functionality for a protein can arise due to the presence of distinct/separate domains to carry out an individual function or upon post-translational modification such as proteolytic cleavage which may result in acquisition of a new function. In the present example Rv1694 has been suspected to possess two entirely diverse activities i.e. haemolytic and RNA methylation activities, which is rather unusual. Since mycobacterium can have a complicated external environment after entry into macrophages, the role of an individual protein like Rv1694 needs a thorough characterization in order to decipher its location specific function during the initial phase of infection. Such studies are important to prove or disprove its diverse/distinct functions to correlate its probable role in pathogenicity of *M. tb*. The present study is aimed at answering the following questions viz., (i) Can this protein be present in two distinct locations and perform two diverse functions? (ii) If yes, any other molecule(s) assist this protein to perform these diverse functions? (iii) How the hemolytic function is orchestrated by it?

The answers to the above questions can be unambiguously obtained only with the help of detailed characterization of Rv1694 as purified Rv1694, *per se*, must exhibit the said activities under *in vitro*, conditions. In this manuscript, we have presented several experimental evidences which clearly show that the Rv1694 has membrane destabilizing activity as well as the RNA methyltransferase activity.

## Results

### Homology of Rv1694 of *M. tb*

**Figure 1:**
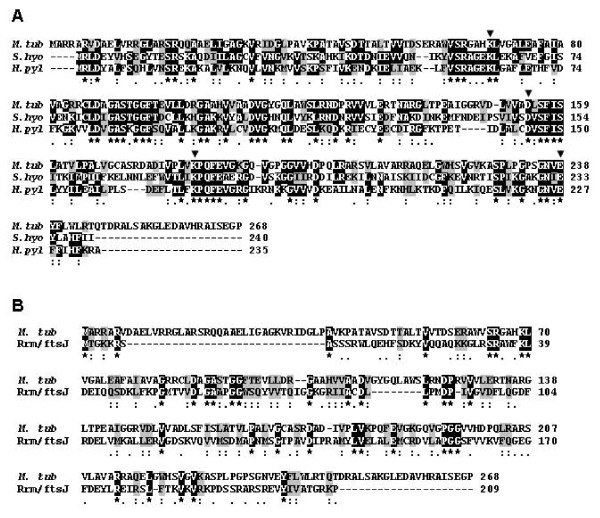
**(A) Multiple sequence alignment of Rv1694 with pore forming hemolysins**. Multiple sequence alignment (BCM search launcher) of Rv1694 (TlyA) of *M. tb *with TlyA sequences of *S. hyodysenteria *and *H. pylori*. TlyA of Mtb shows 58% homology with TlyA of both *S. hyodysenteria *(38% identity) and H. pylori (32% identity). TlyAs of all these bacteria also have K-D-K-E motif (indicated with inverted arrow) (▼) which were predicted to be signature motiffs of ribosomal RNA methyltransferases. **(B) Alignment with ribosomal RNA methyltransferase: **Homology of Rv1694 with ribosomal RNA methyltransferase (FtsJ/RrmJ) from *E. coli *(Multiple sequence alignment with ClustalW). TlyA of Mtb shows 48% homology with FtsJ/RrmJ.

**Rv1694**, *tlyA *gene product of H37Rv, is an alkaline protein that has >40% similarity with other cytotoxins/hemolysins. Initial annotation has referred the *tlyA *gene product as a cytotoxin/hemolysin homologue and it was classified as a hypothetical 'pore forming hemolysin'. The multiple sequence alignment of *tlyA *gene products of other pore forming hemolysins is shown in Figure 1A. For example, the identity amounts to ~38% with *Serpulina hyodysenteriae *and ~32% with *Helicobacter pylori *[8-10]. In addition, the Rv1694 also contains a motifs that align well with rRNA methyltransferase and RNA binding proteins as it displays considerable homology with FtsJ/RrmJ (~48% homology, 23 S rRNA methylase) of *E. coli *as shown in Figure 1B[17,18]. Another common feature seen in the alignment of large RrmJ class was that only four residues, corresponding to the K^38^-D^124^-K^164^-E^199 ^tetrad in *E. coli *RrmJ, are invariant and were common to several families of site specific methyltransferases that modify 2'-hydroxyl groups of ribose moieties of ribosomal RNA. Since Rv1694 also has well conserved K^69^-D^154^-K^182^-E^238 ^tetrad, it has been re-annotated as rRNA methyltransferase [19]. Currently, the National Center for Biotechnology Information, USA has listed the Rv1694 as a member of COG1189 (clusters of the orthologous groups of proteins) which were predicted to be rRNA methylating enzymes. It is note worthy that both hemolysins of *S. hyodysenteria *and *H. pylori *also have the K-D-K-E tetrad (marked with a downward arrow '▼') as shown in Figure 1A. Hence, the *tlyA *gene product exhibits homology with two diverse classes of proteins viz. hemolysin/cytolysin as well as ribosomal RNA methyltransferases.

### Cloning, expression and purification of Rv1694

**Figure 2:**
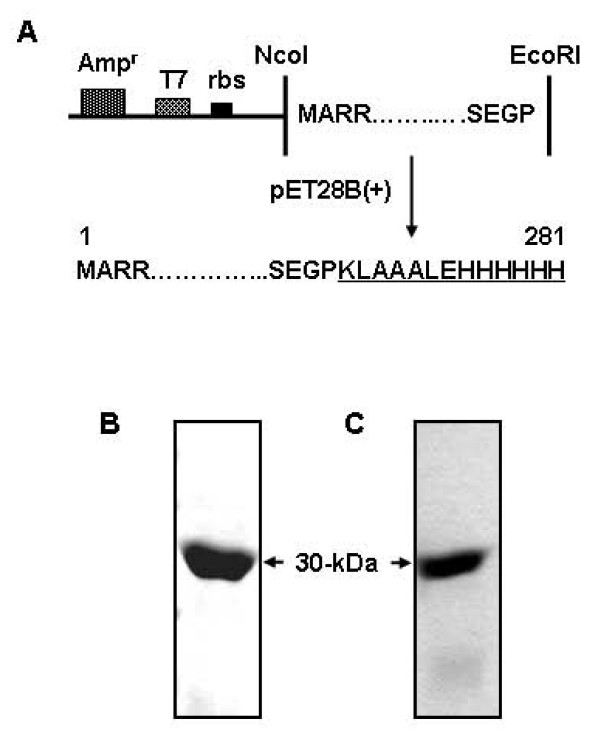
**(A) Cloning of Rv1694**. Cloning strategy of Rv1694 gene in *E. coli *expression vectors, pT7Nc and pET28a(+). The latter vector provides a C-terminal 6-histidine tag. **(B) Purification of recombinant Rv1694: **Expression of 6-histidine-Rv1694 in BL21(DE3) CodonPlus-RIPL *E. coli *after induction with 1 mM IPTG which was purified on Ni-NTA affinity column. Protein sample was electrophoresed on 12% SDS-PAGE and stained with coomassie blue R-250. The purified Rv1694 routinely exhibited >95% purity. **(C) Immuno detection of Rv1694: **Purified Rv1694 was electrophoresed on 12% SDS-PAGE and the immuno-probed with Rv1694 specific immune rabbit serum (at 1:1000 dilution) and the secondary antibody was anti-rabbit IgG HRP- antibody (1:2000 dilution). The specific protein band of Rv1694 can be seen at 30 kDa.

**Figure 3:**
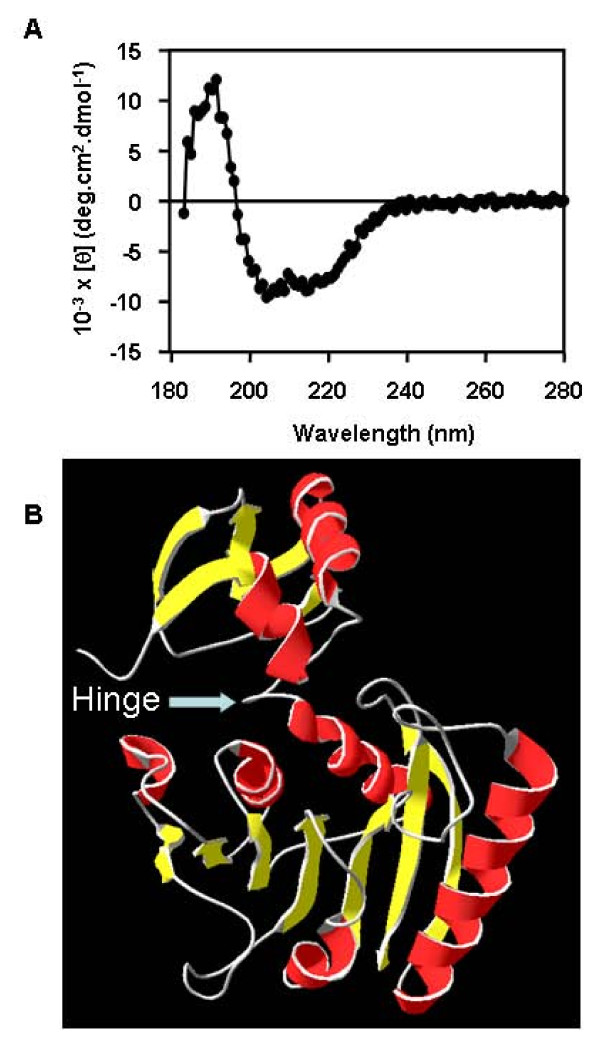
**(A) Circular-Dichroic spectra of Rv1694**. Rv1694-6-histidine tag fusion protein (0.5 mg/ml in 10 mM Na-Phosphate buffer, pH 7.4) was used for obtaining CD spectra. CD data, as interpreted from Yang's calculation, shows that Rv1694 has 23% α-helix, 32.8% β-sheet, 7.7% turns and 36.5% random coil. **(B) Model of Rv1694: **Model of Rv1694 was built using the putative hemolysin from *Streptococcus thermophilus*, using 3HP7.pdb as template with the help of Swiss Model Server [33]. The Rv1694 has a typical fold of a ribosomal RNA polymerase consisting of 7 β-sheets surrounded by 5 α-helices. Please note that the amino acids 246-268(QTD...EGP) are not present in the depicted model. The white arrow points the region containing basic amino acids between the domains susceptible to proteases like trypsin/Proteinase K.

The full-length gene of Rv1694 (828 bp) was cloned in pT7Nc (without 6-his tag) and pET28a(+) (with 6-histidine tag at the carboxy terminus) vectors as shown in Figure. 2A and was expressed in *E. coli *BL21(DE3) CodonPlus-RIPL. The *E. coli *were routinely induced with 1 mM IPTG at 28°C and significant amount of the Rv1694 bearing the 6-histidine tag was isolated from the soluble fraction with the help of Ni-NTA affinity column. The purity was consistently found to be >95% as judged by 12% SDS-PAGE (Figure 2B). The average yield of the purified protein was ~1.0 mg per liter of culture. Figure 2C shows the identification of Rv1694 with immune rabbit serum raised against it. The Circular-Dichroic spectrum of purified Rv1694 indicated the presence of 23% of α-helix, 33% of β-sheet and 37% of random coil (Figure 3A). We have also confirmed the integrity of the protein by tryptic digestion and MALDI-TOFF analysis which was in excellent agreement with data base sequences present in ExPaSyhttp://au.expasy.org/tools/peptide-mass.html (data not shown). The CD data was also consistent with a homology model shown in Figure 3B, which was built with Swiss-Model server using 3hp7A.pdb as template which represents the putative hemolysin from *Streptococcus thermophilus *[20-22].

### Rv1694 has hemolytic activity

**Figure 4:**
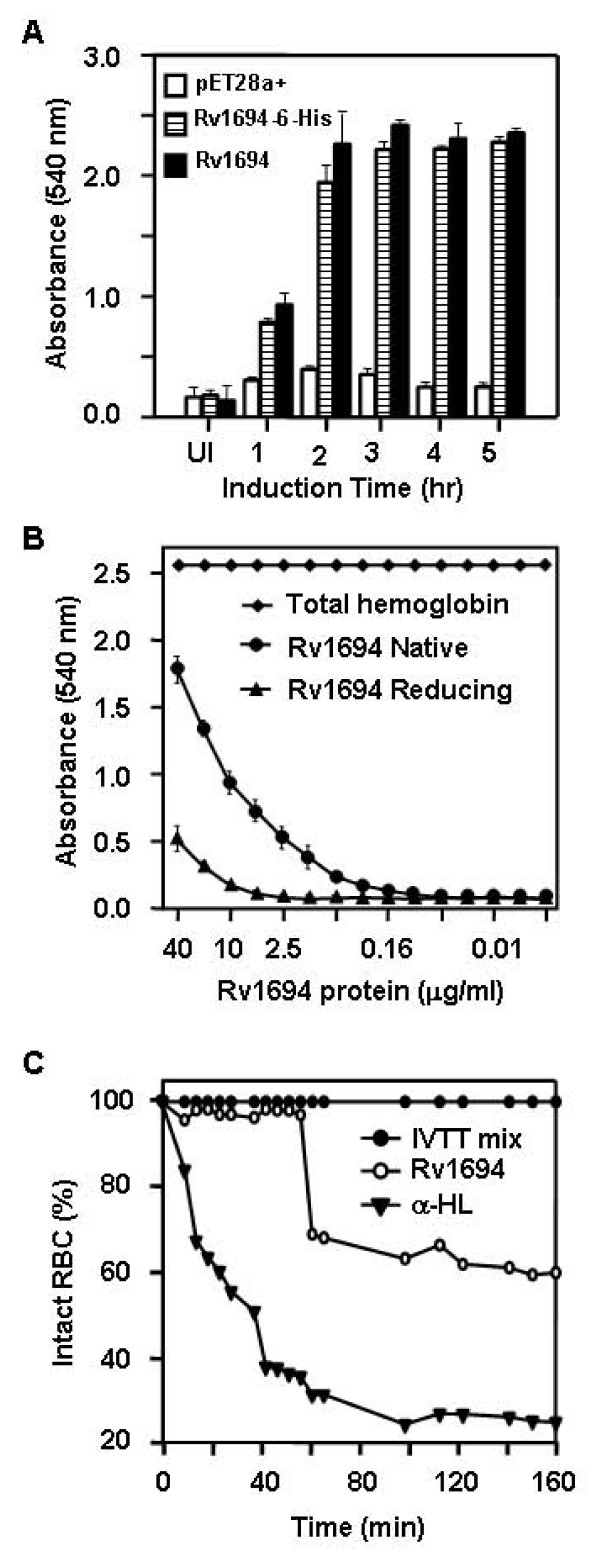
**(A) Contact dependent hemolysis of Rv1694**. Rv1694 (with and w/o His-tag) and mock vector transformed *E. coli *were examined for contact dependent lysis of rabbit RBC. *E. coli *(~10^7^) cells and rabbit erythrocytes (~10^5^) were mixed and briefly centrifuged to ensure close contact between bacteria and RBC. The resultant mixture of cells was incubated at 37°C for 24 to 30 hours. Degree of lysis was monitored by measuring the absorbance at 540 nm of a cell-free supernatant. UI indicates uninduced *E. coli *and groups labeled with 1-5 were harvested time in hour(s) from induction (with 1.0 mM IPTG) time point. Error bars represent standard deviation of two independent experiments. **(B) Hemolytic activity of the purified Rv1694: **Specific hemolytic activity of purified Rv1694 (40 μg/ml) was carried out by two-fold serial dilution of Rv1694 which was mixed with rabbit RBC (1.5%). After 24 hrs of incubation, the absorbance was measured at 540 nm for release of hemoglobin. At ~50% hemolysis, the protein concentration was 18.0 μmug/ml. In presence of thiol-reducing agent, the hemolytic activity was 3.6 fold lesser than the maximum activity of Rv1694. Total haemoglobin release was obtained by lysing the RBC with deionized water. Error bars represent standard deviation from three independent experiments. **(C) Hemolytic activity of Rv1694 generated by coupled *in vitro *transcription and translation: **Rv1694 generated by coupled *in vitro *transcription and translation (~5.0 out of 50.0 μl reaction mix) was mixed with 100 μl of 0.3% rRBC in a 96 well plate as described earlier and the absorbance at 590 nm for rRBC lysis was noted every 10 minutes. As a positive control, we have also translated staphylococcal α-HL and followed its hemolysis.

*E. coli *has no natural homolog of Rv1694, thus, it was not expected to exhibit any contact dependent hemolysis of red blood cells. In order to confirm the hemolytic activity of Rv1694 expressing *E. coli*, we have analyzed both of our constructs (with and without 6-hisitidine tag) for contact dependent haemolytic property. The data in Figure 4A represents the contact dependent hemolysis of rabbit RBCs (rRBC) in comparison to uninduced and mock vector (pET28a+) transformed *E. coli*. Consistent hemolysis of rRBCs was observed in ~30 hrs of incubation time.

We have also examined the specific activity of purified Rv1694, shown in Figure 4B. The amount of Rv1694 needed for 50% hemolysis, in 24 hrs, was ~18.0 μmug/ml while the maximum lysis observable was ~70%. The time taken to achieve hemolysis was rather long but consistent with slow contact dependent hemolysis observed in the published literature mentioned earlier. The slow lysis could be due to slow transfer/assembly of Rv1694 from *E. coli *to RBC membrane, when compared to conventional hemolysins such as Aerolysin or Staphylococcal α-hemolysin which assemble on target cells within minutes as they are secreted from the corresponding bacterium [23,24]. Since, Rv1694 contains two cysteine residues; the role of these Cys residues in the hemolysis was also examined in presence of reducing agents. Interestingly, the hemolytic activity was reduced by ~3.6 fold to that of the non-reduced form as shown in Figure 4B. This observation suggests that the hemolytic activity of Rv1694 is dependent on intact disulfide bond(s).

The above mentioned data on haemolysis, shown in Figure 4B, highlights that the Rv1694 is monomeric protein before binding to the cell membranes and it readily oligomerizes after binding to target membranes. In order to rule out any pre-existent, loose, oligomeric aggregates responsible for the haemolysis of rRBCs in our experiments, we have generated the Rv1694 by *in vitro *transcription and translation (IVTT) and examined for hemolytic property. The data in Figure 4C shows the haemolytic activity of Rv1694 generated by IVTT, which is unambiguous and consistent with the slow hemolysis data shown in Figures 4A and 4B. We have also examined the hemolysis of human RBCs and it was observed that the human RBCs were also susceptible to lysis by Rv1694 (data not shown).

### Visualization of Rv1694 on *E. coli *surface

**Figure 5:**
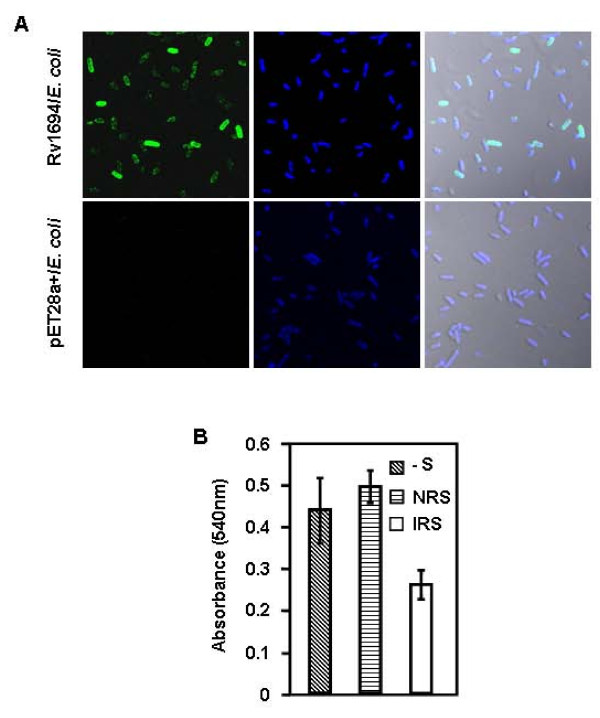
**(A) Immuno-fluorescence localization of Rv1694 on *E. coli***: *E. coli *transformed with Rv1694 or pET28a+ was immunostained with immune rabbit serum followed by detection with Cy2 conjugated goat anti-rabbit IgG (green fluorescent). Left side of the panels show the Rv1694 localization (Green), middle panels show the DAPI stained bacteria (Blue) and right panels show the merged images. **(B) Inhibition of contact dependent hemolysis by Immune rabbit serum: **Rv1694 transformed *E. coli *was incubated with and without pre-immune/immune rabbit serum as described for Figure 4A. Error bars represent standard deviation of two independent experiments.

Since Rv1694 expressing *E. coli *showed contact dependent hemolysis, it was important to localize by staining the *E. coli *expressing the Rv1694 with rabbit antiserum specific to it. The confocal microscopic pictures of *E. coli *stained for the Rv1694 are shown in Figure 5A. Strikingly, the Rv1694 was present on the outer cell wall of *E. coli *as seen from the specific staining of the polyclonal antibody raised by us. It is important to note that neither mock transformed *E. coli *(stained with Rv1694 specific immune serum) nor secondary antibody used in the study showed any staining. These vital controls unambiguously prove the presence of Rv1694 on the cell wall.

We next examined whether the immune serum of Rv1694 can inhibit the contact dependent hemolytic activity of *E. coli *expressing the Rv1694. As shown in Figure 5B, the hemolysis was reduced by about 50% in comparison to the hemolysis in the absence of immune serum. The observed inhibition of hemolysis by the immune serum was specific as pre-immune serum of the same rabbit had no effect on the contact dependent hemolytic activity of the *E. coli *expressing the Rv1694. These observations suggest that the Rv1694 was expressed on the cell wall of *E. coli *and the anti-Rv1694 antibody neutralizes the contacts needed for the assembly and lysis.

### Binding and oligomerization of Rv1694 on RBC membranes

**Figure 6:**
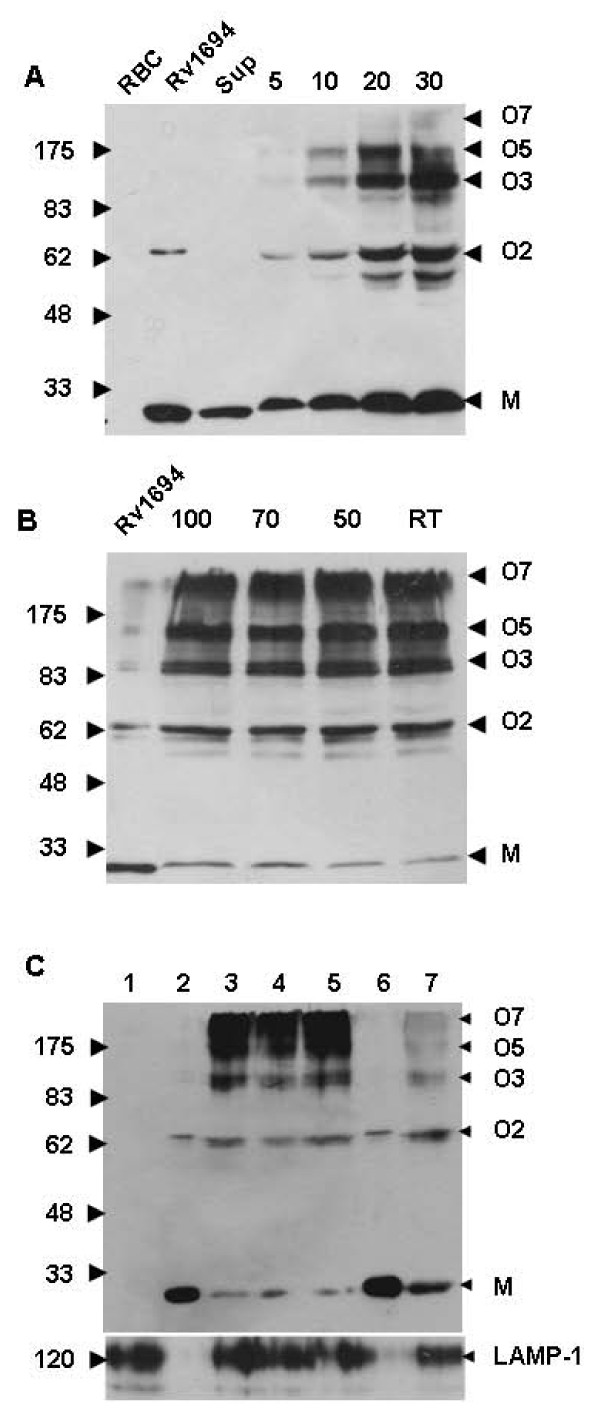
**(A) Binding and oligomerization of Rv1694 on rRBC membrane**. Various amounts of purified Rv1694 were incubated with 2% rRBC for 30 min and the resultant blot was immuno probed with anti-6-histidine-antibody. Lanes indicated with RBC, Sup and numbers respectively indicate rRBC membrane, purified Rv1694, supernatant of rRBC, rRBC incubated with 5.0 μg, 10.0 μg, 20.0 μg and 30.0 μg Rv1694 respectively. **(B) Oligomerization of Rv1694 on RBC membrane in the absence of reducing agents: **Oligomerization of Rv1694 (10 μmug) on rRBC (2%) was carried out as described in methods section. The samples were washed and dissolved in 4% SDS and 5× laemmli sample buffer without β-mercaptoethanol and immuno probed with anti-6-histidine-antibody. Lane 1. Rv1694 purified protein; Lanes 2-5 show the oligomers of Rv1694 on the RBC membrane at indicated temperatures (°C). **(C) Binding and oligomerization of Rv1694 protein on the Phagosomal membrane: **Aliquots were taken out at indicated time points from the Rv1694 incubated phagosomal preparations, electrophoresed on 8% SDS-PAGE (non-reducing), immuno probed with anti-6-histidine-antibody. Lane 1**: **Phagosomal preparation alone; Lane 2**: **Unboiled Rv1694; Lanes 3 to 5**: **Unboiled samples of phagosomal preparations and Rv1694 incubated for 10.0, 30.0, and 60.0 min respectively. Lane 6**: **Boiled Rv1694 protein; Lane 7**: **Boiled sample of lane 5. Labels viz., M, O3, O5, O6 and O7 indicate monomers, trimers, pentamers, hexamers and heptamers respectively. The blot was stripped and re-probed with anti-LAMP-1 antibody to ascertain the phagosomal preparation. The data shown is one of the three independent experiments.

We further examined whether or not Rv1694 can form ordered oligomers which must be present and responsible for the slow but consistent hemolysis observed by us. Hydropathy plot analysis of Rv1694 primary sequence has revealed that there are two probable transmembrane regions viz. amino acids 67-87 and 150-170. To confirm the binding and oligomerization of Rv1694 to RBC membranes, we have incubated the Rv1694 with rRBCs, isolated the membrane fragments and visualized its oligomeric forms by SDS-PAGE. The Rv1694 showed strong binding to rRBC membranes and formed stable oligomers which were easily detectable with anti-6-histidine antibody (Figure 6A). The oligomers of Rv1694 showed concentration dependency and the kinetics of oligomerization on rRBC membranes was found to be slow in comparison to well studied, conventional hemolysins.

The data shown in Figure 6A and 6B lets us infer that the Rv1694 can form oligomers ranging from dimers, to heptamers based on the mobility identified with anti-6-histidine antibody. The oligomers formed on RBC membranes were found to be resistant to temperatures up to 100°C and 4% SDS very much like the oligomers of staphylococcal α-hemolysin (although very slight dissociation was found at 100°C, but that was relatively insignificant), but significant dissociation of the oligomer was observed in the presence of reducing agent. This result suggests that the hemolytic activity of Rv1694 was due to the formation of stable oligomers on target cell membranes only and the intermolecular disulfide bond may be necessary for the oligomerization.

### Rv1694 binds to and oligomerizes on macrophage phagosomal membranes

Earlier studies have suggested that the *M. tuberculosis*, after entry into macrophages, appears to compromise the integrity of phagosomal membranes for its survival. To understand any possible role for Rv1694 in this process, we have examined the binding and oligomerization of Rv1694 on phagosomes isolated from RAW264.7 cells for different time periods i.e. 10, 30, 60 min. In Figure 6C, the presence of LAMP-1, 120 kDa phagolysosome glycoprotein protein marker, authenticates our phagosomal preparation. The Rv1694 showed strong binding to the phagosomal membranes and formed stable oligomers, which are very similar to the ones seen on rRBCs including their stability in 4% SDS and susceptibility to near boiling temperatures under non-reducing conditions. Moreover, unboiled samples prove the absence of any pre-existing oligomer of Rv1694 added to phagosomal preparation. In summary, all these observations suggest that the Rv1694 has the ability to bind to target membranes such as phagosomal membranes and can form multimeric structures for destabilization. In this process it appears that a disulfide bond seems to play a key role.

### Limited proteolysis of Rv1694

**Figure 7:**
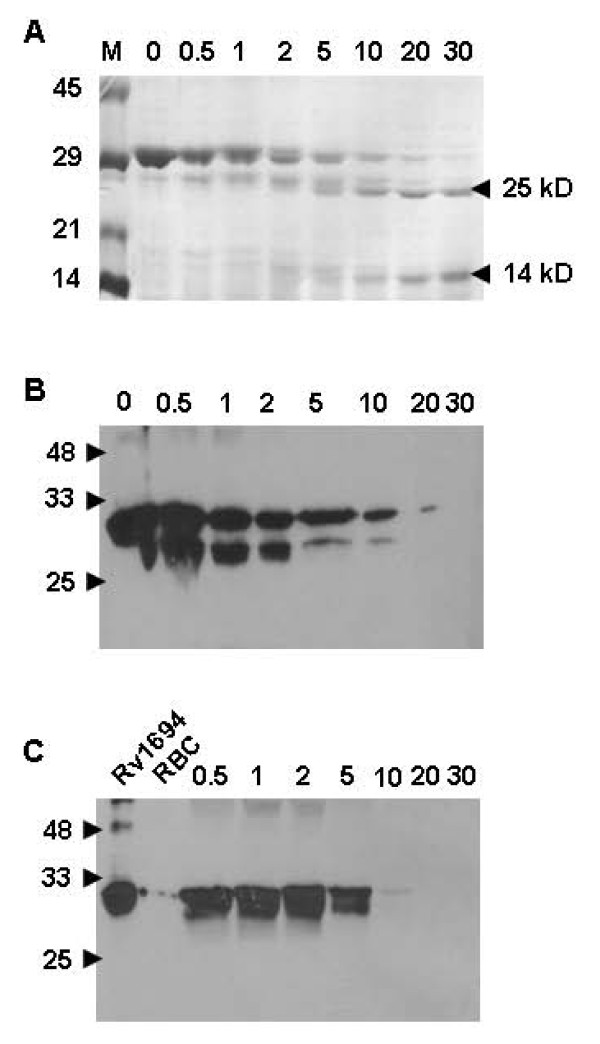
**(A) & (B) Limited proteolysis of Rv1694 with Proteinase K**. Rv1694 was partially digested with Proteinase K for indicated times, electrophoresed on 12% SDS-PAGE, stained with coomassie brilliant blue R-250. The lanes were marked to indicate M for protein molecular weight markers, numbers represent the time points: 0 for Rv1694 protein only and lanes marked with 0.5, 1, 2, 5, 10, 20 and 30 respectively represent Proteinase K treatment times in minutes. The arrow on the right indicates the stable domains of Rv1694. The panel **(B) **was obtained by probing with anti-6-histidine monoclonal antibody. **(C) Limited proteolysis of Rv1694 bound to rRBC membranes: **Purified Rv1694 protein and rRBC were incubated for 30 min and subjected to digestion with Proteinase K for indicated periods of time. At the end of the incubation, the rRBC were mixed with 2 mM PMSF, washed twice with hypotonic buffer, solublized the RBC membranes and were electrophoresed on 12% SDS-PAGE, probed with anti-6-histidine-monoclonal antibody. The lanes marked with Rv1694 and RBC respectively represent Rv1694 protein only and rRBC membrane only. Lanes indicated with 0.5, 1, 2, 5, 10, 20 and 30 respectively represents the Proteinase K treatment times. Results shown are one of the two independent experiments.

Limited proteolysis provides valuable information regarding the existence of stable domains of proteins/regions and also susceptible regions that might be protected against proteolysis upon interaction with other proteins or cell membranes. Keeping this in mind, we have digested the Rv1694 with Proteinase K in presence and absence of membrane for different time periods and examined the resultant protein fragments by SDS-PAGE. The SDS-PAGE gels were stained with Coomassie Brilliant blue and also by immuno blotting with anti-6-histidine antibody. As shown in Figure 7A, after 30 min of Proteinase K digestion, there were two prominent bands of size around 25 kDa and 14 kDa, which seem to be resistant to further digestion as seen with Coomassie brilliant blue staining. However, the cleavage pattern of Rv1694, shown in Figure 7B, obtained with anti-6-histidine antibody suggests that the amino terminal of the Rv1694 was more susceptible to proteases such as Proteinase K. It should be noted that any cleavage at the carboxy terminus cannot be detected with the anti-6-histidine antibody. Limited proteolysis of Rv1694, bound to the RBC membrane, has yielded the pattern suggestive of inaccessibility of the amino terminus as we could see the intact protein in the shorter time frames (Figure 7C). The proteolytic patterns were also consistent with the model of Rv1694 shown in Figure 3B. As shown in Figure 3B, the two visible domains of Rv1694 viz. N-terminal domain consisting of 1 to ~54 amino acids and the second domain of 67 to 281 amino acids are connected by amino acid segment with two Arg residues, which are generally susceptible to trypsin or Proteinase K like enzymes. If a protease nicks the region between two domains, it should result in a domain that is smaller by 5 kD i.e. ~25 kD (molecular weight of Rv1694 with 6-histidine tag is 30 kD). In summary, the amino-terminal of the Rv1694 protein was more accessible to the Proteinase K in the soluble state that becomes inaccessible when bound to the target membranes.

### Rv1694 is a ribosomal RNA methylase

In order to evaluate the ribosomal RNA methyltransferase activity of Rv1694, we attempted several approaches as described below:

### Capreomycin inhibits the growth of *E. coli *transformed with Rv1694

**Figure 8:**
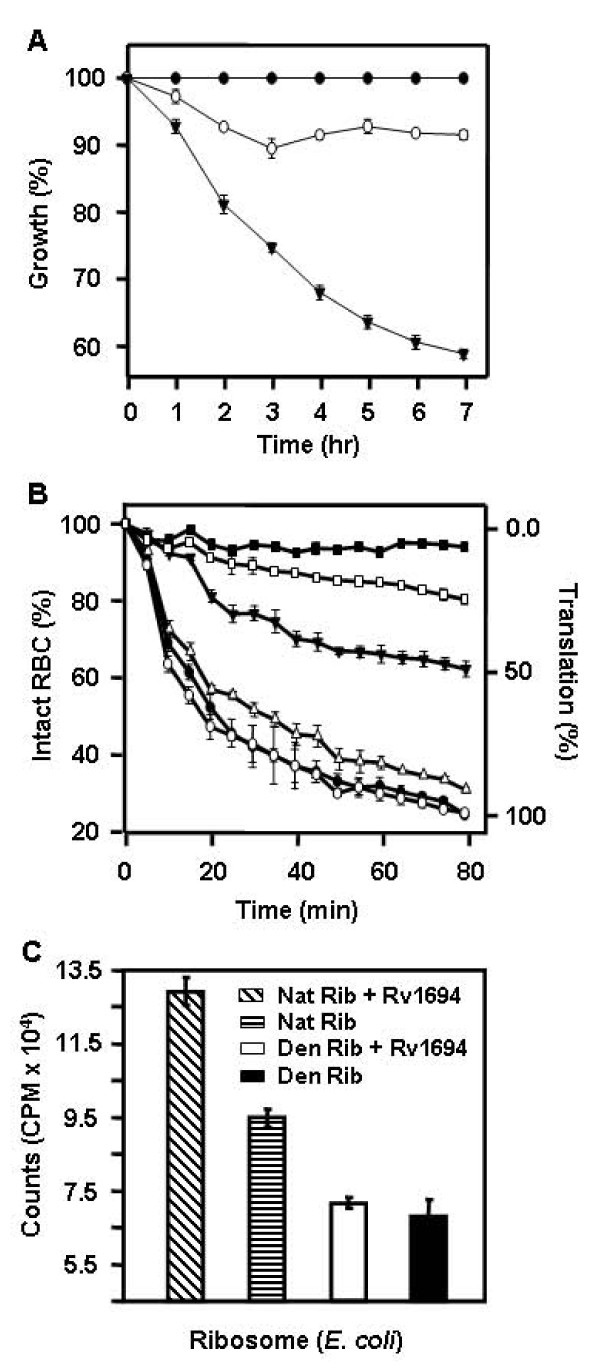
**(A) Growth of *E. coli *expressing Rv1694 in presence of capreomycin**: Growth of *E. coli *expressing Rv1694 in the absence (●) and presence (▼) of capreomycin (100 μmug/ml), and pET28a+ transformed *E. coli *(○). Error bars represent standard errors from two independent experiments. **(B) Inhibition of coupled *in vitro *transcription-translation by capreomycin: **S30 extracts of Rv1694 or pT7Nc transformed *E. Coli *BL21(DE3) were prepared and *in vitro *transcription and translation reactions were carried out with either 16 or 80 ng/ml capreomycin. The hemolytic activity of staphylococcal α-hemolysin (used as a reporter) was monitored at OD_595_. All points for all curves were calculated with respect to 100% lysis of rRBCs and average of three independent experiments is shown. Various symbols represent (●) Rv1694 without capreomycin; (○) mock vector without capreomycin; (▼) Rv1694 at 16 ng/ml capreomycin; (∆) mock vector at 16 ng/ml capreomycin; (■) Rv1694 80 at ng/ml capreomycin; (□) mock vector at 80 ng/ml capreomycin. **(C) *In vitro *methylation activity of purified Rv1694 protein on isolated Ribosome: **Methylation of *E. coli *ribosomes in presence or absence of purified Rv1694 and [^3^H]S-adenosylmethionine. From left, Crossed pattern bar represents the reaction mix with native ribosome ('Nat Rib') and Rv1694 ('Prot'), horizontal pattern bar represents reaction mix with native ribosome only, open bar represents reaction mix with heat (65°C) denatured ribosome ('Den Rib') and Rv1694 and filled bar represents reaction mix with denatured ribosome only. Error bars represent standard errors from triplicate experiments.

In our first approach, we monitored the growth rates of *E. coli *expressing the Rv1694 and mock vector (pET28a+) transformed *E. coli *without or with capreomycin (100 μmug/ml) as shown in Figure 8A. In the presence of capreomycin, the mock vector transformed *E. coli *growth was retarded by only ~10% while the *E. coli *expressing the Rv1694 construct did not grow efficiently (> 40% loss). We also ensured that these cultures expressed the Rv1694 by examining for hemolysis at each time point for the data plotted (data not shown). This experiment confirms the Rv1694 mediated methylation of ribosomal RNA of *E. coli *under *in vivo *conditions, as the mock vector transformed control has practically showed no dramatic retardation of growth in the presence of capreomycin.

### Capreomycin inhibits *in vitro *translation of S30 extract of Rv1694 transformed *E. coli*

In the second approach, we also analyzed the *in vivo *methylation activity of Rv1694 by coupled *in vitro *transcription-translation with capreomycin. In this experiment, we prepared the S30 extracts of *E. coli *expressing the Rv1694 along with controls. The S30 extract was supplemented with all necessary components to monitor the translation of the S30 extract in the presence and absence of capreomycin. We have used the supercoiled plasmid of α-hemolysin of *Staphylococcus aureus *as a reporter to examine the translational activity of the S30 extract as staphylococcal α-hemolysin is very efficient in inducing the lysis of rRBCs. Also, the kinetics of hemolysis of α-hemolysin was well established and very fast in comparison to the kinetics of lysis of Rv1694 [25]. Moreover, the assay is fast and reliable as the efficiency of translation by way of lysis of RBCs can be compared with the values reported in the literature and does not require any specialized equipment or reagents.

We have carried out the *in vitro *transcription and translation experiments at two different concentrations of capreomycin (16 ng/ml and 80 ng/ml), in order to closely match the observations reported earlier [15]. In Figure 8B, in the absence of capreomycin, the translational efficiency of S30 extracts of Rv1694 expressing *E. coli *and mock vector *E. coli *showed nearly identical translational efficiency of the staphylococcal α-hemolysin reporter (filled circles vs. open circles) and this value was taken as 100% translation efficiency. At 16 ng/ml capreomycin, the S30 extract made from Rv1694 expressing *E. coli *showed reduced translation (~50% loss) i.e. considerable loss of hemolysis (solid inverted triangles) where as the mock vector transformed *E. coli *showed no loss of translation (open triangles). At 80 ng/ml capreomycin concentration, the S30 extract made from Rv1694 expressing *E. coli *showed no translation of reporter (solid squares), whereas the vector transformed *E. coli *showed about 26% lysis (solid squares vs. open squares) compared to the translation without capreomycin. It should be noted that the *E. coli *is partially sensitive to capreomycin at higher concentrations i.e. >80 ng/ml. This experiment, once again, confirms the ribosomal RNA methylation activity of Rv1694.

### *In vitro *methylation activity of Rv1694 on ribosome

In addition to capreomycin sensitivity assays described above, we have also examined the incorporation of radioactive methyl group of S-adenosylmethionine ([^3^H]-SAM) into the ribosomal RNA by Rv1694. In this assay, we have used crude ribosomes as substrate for Rv1694 in the presence of the methyl group donor ([^3^H]-SAM) and judged the extent of methylation reaction. Assay was carried out with and without Rv1694 and ribosomes under native and heat denatured conditions. It was clear from Figure 8C that the native ribosome, in the presence of purified Rv1694, has 36.3% more methylation in comparison to ribosomal preparation alone. However, heat denatured ribosomes, in the presence of purified Rv1694, didn't show any significant incorporation of the methyl group of [^3^H]-SAM in comparison to the heat denatured ribosomes alone. This result, once again, confirmed that the purified Rv1694 was also ribosomal RNA methylase and it transfers the methyl group from S-adenosylmethionine to rRNA.

## Discussion

The aim of the present study is to decipher the other face of mycobacterial Rv1694 which has been attributed with ribosomal RNA methylation activity.

The data presented in Figures 4, 5, 6, 7, which assessed the activity of Rv1694 under *in vitro *conditions, unequivocally, confirm the other feature of Rv1694 i.e. its membrane destabilizing attribute. Firstly, the *E. coli *harbouring the *tlyA *has clearly exhibited hemolysis much like the mycobacterial strain studied earlier. This observation also implies that the cellular localization of Rv1694 is nearly same in both *M. tb *and *E. coli *which may involve common transport mechanism. Noteworthy feature of our confocal data was that the Rv1694 was exported to the cell wall of *E. coli *despite the absence of any signal sequence. Secondly, the purified protein also exhibited unambiguous hemolysis under *in vitro *conditions (Figure 4A) which was sensitive to the presence of reducing agents. The hemolytic activity of the purified protein was reduced drastically in the presence of reducing agents indicating the need for the presence of an intact disulfide bond. Moreover, Rv1694 irreversibly binds to and oligomerizes on red blood cell membrane and was resistant to heat and SDS very much like the conventional hemolysins but unlike them, the oligomers of Rv1694 dissociate in the presence of thiol-reducing agents. While reducing agents abolish the activity by 90% and the residual activity seen in Figure 4A could be due to the presence of higher order oligomers (around pentamers) which were found to be un-dissociable. Unfortunately, these oligomers were rigid and did not fly well in MALDI-TOFF spectrometers for deeper analysis. Moreover, consistent with the haemolytic nature, the data in Figure 6B and Figure 6C revealed the Rv1694's ability to bind to and oligomerize on isolated phagosomal membranes also. This oligomerization was once again sensitive to reducing agents. Our limited proteolysis data also revealed that the amino terminus was more exposed to proteases in solution while it was found to be inaccessible when it was membrane bound. Hence, a reasonable working model for the contact dependent haemolytic property of *E. coli *expressing the Rv1694 could be due to contacts between *E. coli *and target membranes through the insertion of its amino terminus in the target membrane while its carboxy terminus could be anchored to host cell wall. Overall, the hemolytic property of this protein was due to ordered assembly of this protein on target cell membranes through its hydrophobic pockets than due to any pre-existing aggregate(s) as the Rv1694, generated by *in vitro *transcription and translation, also exhibited unambiguous hemolysis. Our data also rules out the role of any unidentified protein complexing with the Rv1694 for the observed haemolytic activity as the oligomers seen in our SDS-PAGE gels were composed of homo-oligomers of Rv1694 as the molecular weights of the oligomers were consistent with the multimeric forms of Rv1694. In support of this, the SDS-PAGE of oligomers, after boiling in presence of reducing agents (to dissociate the oligomers) has yielded the corresponding monomeric Rv1694.

In general, more than one third of bacterial pore forming toxins act as common virulence factors for the host pathogenic bacteria. These toxins have two distinct states: a water soluble form before oligomerization and a transmembrane form after oligomerization and conversion between these two states do not require any assistance from chaperones but require a hydrophobic *milieu*. On the other hand the data presented here on Rv1694 is interesting as it does not have any classical signal sequence like the conventional secreted forms of hemolysins. In this regard, the data reported by Bloom's group had highlighted this possibility. These authors have shown that the live BCG organisms were able to orchestrate phagosomal membrane permeability which can allow uptake of molecules upto 70 kD [26]. This observation suggests the role of a hemolysin like or membrane destabilizing molecule after successful entry into macrophages. The need for the association of Rv1694 with phagosome compartments is also important in the context for understanding the MHC class I presentation which requires antigen localized to the cytoplasmic compartment of antigen-presenting cells. It has been shown that the human CD8+ T cells recognized epitopes of a 28-kD hemolysin (sequences of Rv1694) from HLA*0201+ persons with latent tuberculosis infection [27]. Hence, the best role that can be visualized for the Rv1694 would be limited to destabilizing the phagosomal compartments for which we have shown the evidence in Figure 6C. For this activity, it is essential that the Rv1694 must be present at or beneath the surface of mycobacterial cell envelope. Upon contact/entry with the host macrophage or a phagosomal compartment, it can attach to the membrane for destabilization. The contact dependent hemolysis observed by us can indirectly suggest this possibility.

In addition to the haemolytic activity of Rv1694, our preparations have shown an unambiguous ribosomal RNA methyltransferase activity as well. Expression of Rv1694 had resulted in susceptibility to capreomycin (Figure 8). All these observations together summarily suggest that the Rv1694 is dually active protein.

## Conclusions

In summary, we have been successful in providing clear answers to the questions mentioned earlier viz. (i) The Rv1694 can be present in two distinct locations viz. intracellularly to methylate the ribosomal RNA which confers susceptibility to capreomycin and also at the cell membrane to destabilize the target membranes upon its contact. (ii) Based on the oligomeric bands and the dissociation of the oligomers under reducing, boiling conditions confirm that the Rv1694 might not be assisted by any other protein (iii) The orchestration of hemolysis by this protein involves a possible burial of its amino terminus in the target membranes upon its contact while its carboxy terminus can anchor with the host bacterial cell wall. To the best of our knowledge, a dual activity has not been demonstrated either for cytolysins or for RNA methyltransferases using highly purified preparations of proteins. Further studies are underway to know the role of this protein in the context of bacterial growth and survival to know the functions of Rv1694.

## Methods

### Materials

*In vitro *transcription and translation kit for *E. coli *T7 S30 Extract System for Circular DNA was purchased from Promega Corporation, USA. *E. coli *BL21(DE3) CodonPlus-RIPL was from Stratagene, USA. Anti-6-histidine mouse IgG HRP labeled antibody (Clone F24-796) from BD Biosciences, USA. Protease inhibitor cocktail and Capreomycin was obtained from Sigma Aldrich, USA. [Methyl*-*^3^H]-*S*-Adenosyl-L-methionine was purchased from American Radiolabeled Chemicals, Inc., USA. All animal experiments were performed after obtaining necessary permission from Institutional Animal Ethics Committee.

### Cloning, expression and purification of Rv1694

Rv1694 was cloned by PCR amplification from the genomic DNA of *M. tb *strain H37Rv (01.Rv.2.10.17.x.DNA). This gene was amplified by using gene specific primers (forward primer, 5'-TATATATccatggCTC-GACGTGCCCGCG and reverse primer, 5'-TATATAgaattcTTACGGGCCCTCGCTAATCGC) and inserted into pT7Nc expression vector made in our laboratory [25]. The recombinant Rv1694 gene was confirmed by DNA sequencing. Rv1694 gene was sub-cloned in pET28(a+) (Kan^r^) expression vector between *Nco*I and *Hind*III, which provides a carboxy terminal 6-histidine-tag for purification by Ni-NTA resin. pET28(a+)-Rv1694 construct was transformed in the BL21(DE3) CodonPlus-RIPL *E. coli *cells. A well isolated, independent colony was inoculated in 10 ml Luria Bertani (LB) medium containing kanamycin (30 μmug/ml) and chloramphenicol (34 μmug/ml) and incubated at 37°C over night. The over night culture was inoculated (1%) into 500 ml LB at 28°C until OD_600 _reached 0.30 to 0.35. The culture was then induced with 1 mM IPTG and was further grown for another 6 hrs at 28°C. The cells were harvested by centrifugation and the pellet was re-suspended in buffer A (50 mM Tris-HCl, pH 8.0 and 150 mM NaCl) containing 1 mg/ml lysozyme, 50 μmul protease inhibitor cocktail per gram bacterial pellet, 1 mM PMSF and incubated on ice for 30 min. The cells were sonicated and the lysate was centrifuged at 30,000 × g for 20 min at 4°C. The supernatant was applied on a pre-equilibrated Ni-NTA column and washed extensively with 10-15 column volumes of equilibration buffer A containing 10 mM imidazole. The column was then washed with five column volumes of 50 mM and 75 mM imidazole containing buffer A. Bound His-tag fusion Rv1694 protein was eluted with 200 to 250 mM imidazole containing buffer A. The purity of the eluted Rv1694 was examined by 12% SDS-PAGE and concentration was estimated by the Bradford's method [28,29].

### Measurement of CD spectra of Rv1694

CD spectra of the native Rv1694 were recorded on a JASCO-815 spectro-polarimeter, at 25°C, in the far UV range of 180-280 nm at scan speed 50 nm/min with a response time of 1 s and slit width 1 nm. A rectangular quartz cell of 1 mm path length was used. All measurements were made at a protein concentration of 0.5 mg/ml. For each spectrum, fifteen scans were collected and the averaged spectra were used for further analysis. Measurements were made in 10 mM phosphate buffer pH 7.5 and buffer scans recorded under the same conditions were subtracted form the protein spectra before further analysis.

### Contact dependent hemolysis

Contact-dependent lysis of rabbit erythrocytes was carried by employing a minor modification of the procedure published earlier [11]. Rv1694 and control vector transformed *E. coli *were grown in LB media (30 μg/ml kanamycin and 34 μg/ml chloramphenicol) at 28°C till the OD_600 _reached 0.3 to 0.35. The culture was induced with 1 mM IPTG and harvested at different time points. The bacterial pellet was resuspended in 25 mM Sodium Phosphate buffer, pH 7.4 and 150 mM NaCl and washed twice with the same buffer. For contact dependent lysis, *E. coli *expressing the Rv1694 or the control vector (~10^7^) were mixed with rabbit erythrocytes (~10^5^) in 1 ml same buffer and centrifuged to ensure close contact between bacteria and red blood cells. For contact dependent lysis with normal and immune rabbit serum against Rv1694, we incubated 1:100 dilution of serum with *E. coli *(10^6^) for 30 min at 25°C prior to incubation with rRBCs at 37°C for 24 to 30 hours. Release of hemoglobin from erythrocytes in supernatant was estimated by measuring the absorbance at 540 nm.

### Immuno-labelling of *E. coli *with Rv1694 specific immune rabbit serum

Rabbit polyclonal antibody was raised against purified Rv1694. Rv1694 and mock vector transformed *E. coli *were harvested at late log phage, washed twice and re-suspended in PBS. Bacteria in PBS suspension was incubated with 1:100 dilution of Rv1694 immune rabbit serum for 1.0 hour at room temperature and washed thrice with PBS. Bacteria were resuspended in PBS and incubated with 1:200 dilution of Cy2 conjugated goat anti-rabbit IgG (Jackson Immuno Research Laboratories, West Grove, PA, USA) for 45 min and with DAPI with 2-3 min, washed thrice with PBS. A thin smear of PBS suspended bacteria was made on the glass slide for observation with a confocal microscope.

### Coupled *in vitro *transcription and translation (IVTT)

*In vitro *translation of super coiled Rv1694-pT7Nc was essentially carried out as described earlier by using *E. coli *T7 S30 extract for circular DNA [25]. To inhibit endogenous RNA polymerase, we added 1 μmul of a 50 μmug/ml solution of rifampicin in 50 μl reaction volume prior to the addition of the DNA template to the reaction mixture.

### Hemolysis assays of purified and IVTT generated Rv1694

Purified Rv1694 (20-40 μg/ml) was two fold serially diluted in 25 mM Sodium Phosphate buffer, pH 7.4 and 150 mM NaCl buffer, mixed with 1.5% rabbit red blood cells (rRBC) or human red blood cells (hRBC) and incubated at room temperature (25°C) for 24 hrs. The absorbance of the supernatant at 540 nm was measured. IVTT generated protein (5 μl) was mixed with 100 μl of 0.3% rRBC in a 96 well plate and the absorbance at 590 nm for the RBC lysis was noted at every 10 min time interval.

### RBC membrane binding assay

Rv1694 (15.0 μmug or as mentioned) was mixed with 2% of the rabbit RBC in 25 mM Sodium Phosphate buffer, pH 7.4 and 150 mM NaCl buffer and incubated at room temperature at 37°C for 30 min (or for indicated time). At the end of the incubation period, the RBCs were pelleted at 200 × g, re-suspended and washed in hypotonic buffer (10 mM Tris-HCl, pH 7.5, 5 mM EDTA, 1 × protease inhibitor cocktail and 1 mM PMSF). RBC membranes were collected by centrifugation (10,000 × g) and solublized with/without β-mercaptoethanol in 4% of SDS at 100°C (or as indicated) for 5 min and electrophoresed on 8% SDS-PAGE. The resolved proteins were transferred to nitrocellulose membrane and probed with anti-6-histidine antibody, followed HRP labelled conjugate.

### Phagosome preparation from RAW 264.7 cell line

RAW 264.7 mouse macrophage-like cells were grown in DMEM with 10% FCS. The cells were washed with PBS, pH 7.4, detached by scraping, resuspended in buffer (0.25 M sucrose and 10 mM HEPES, pH 7.4) containing 1.0 mM phenylmethylsulfonyl fluoride (PMSF), protease inhibitor cocktail, and homogenized. Intact cells and nuclei were removed by centrifugation (200 × *g *for 10 min). The supernatant was centrifuged at 1900 × *g *for 10 min to pellet the crude phagosome and the pallet was washed twice with PBS by centrifugation at 10,000 × g for 5 min at 4°C [30,31]. The phagosome pallet was stored at -80°C if not used immediately.

### Binding and oligomerization on phagosomal membranes

Rv1694 (15.0 μmug) was mixed with phagosomal preparation containing 1.0 mM PMSF and protease inhibitor cocktail and incubated on ice. Aliquots of 20.0 μmul after each time point was taken out and washed twice with PBS, pH 7.4, pallet was mixed with sample loading dye without β-mercaptoethanol containing 4% SDS. Unboiled and boiled samples were electrophoresed on 8.0% SDS-PAGE and probed with anti-6-histidine antibody. Same blots were stripped (100 mM Glycine-HCl, pH 2.5, 20 mM Magnesium acetate and 50 mM KCl) and re-probed with anti-LAMP-1 antibody (1D4B, BD Biosciences, USA) and secondary goat anti-rat IgG HRP labeled antibody (sc-2006, Santa cruz biotechnology, inc., CA, USA).

### Limited Proteolysis

Rv1694 was subjected to digestion with Proteinase K at 25°C by keeping the Rv1694: Proteinase K ratio 50:1 (in solution) and 50:4 with 2% rRBC (for the membrane bound Rv1694). At indicated time points, 10 μg equivalent of the digested protein was taken and inactivated by the addition of 5× Laemmli sample buffer containing 2 mM PMSF. For proteolysis of Rv1694 on the membrane, equal volume of Proteinase K treated protein-rRBCs mix were taken out at each time interval and washed twice with hypotonic buffer (with 2 mM PMSF) to remove unbound proteins and released hemoglobin. The RBC membranes were solublized with 4% SDS at 50°C for 5 min. Both the samples (in solution and on rRBC membrane) were boiled at 100°C for 5 min and electrophoresed on 12% SDS-PAGE.

### Capreomycin sensitivity assays

Bacterial culture was grown in LB media with appropriate antibiotic at 28°C and split into 2 parts at OD_600 _~0.3, and further grown with and without capreomycin (100 μg/ml). After every hour, a small aliquot of culture was withdrawn and OD_600 _was measured for the growth analysis.

### Preparation of S30 extracts

S30 extract was prepared by growing recombinant Rv1694 expressing *E. coli *in LB media in presence of appropriate antibiotic at 28°C and induced with 100 μmuM IPTG at OD_600 _of ~0.35. The bacteria were harvested after 3 hrs. Cells were subjected to ultrasonic disruption (40% amplitude, 10 times, pulse of 10 sec on/off) in buffer (10 mM Tris-HCl, pH 7.4, 10 mM MgCl_2_, 60 mM NH_4_Cl, 6 mM 2-mercaptoethanol), and centrifuged for 30 min at 30,000 × *g *to obtain the supernatant [32]. *In vitro *translation activity was measured in the absence and presence of capreomycin at two concentrations (16 and 80 ng/ml). We have used α-hemolysin from *Staphylococcus aureus *as a reporter gene for the quantitating the efficiency of translation of the ribosomal preparation. Lysis of rRBC, monitored at OD_595_, by the *in vitro *generated α-hemolysin was the direct measure of the translation efficiency of the ribosomes in presence of capreomycin [25].

### *In vitro *methylation of ribosome

Ribosomes were prepared by ultracentrifugation of S30 extract of *E. coli *at 150,000 ×g for 1.0 hour at 4°C, as described above. Methylation assay was carried out with native and heat denatured ribosomes (at 65°C for 5.0 min). Reaction mixture contained 50 mM Tris-HCl, pH 7.4, 3 mM Mg(OAc)_2_, 200 mM NH_4_Cl, 5 mM dithiothreitol, 1.5 μmuM [Methyl-^3^H]- Adenosyl-L-methionine (81 Ci/mmol), 18.0 μmug ribosome and 4.0 μmug purified Rv1694 protein in a total volume of 25.0 μmul. Incubation was carried out at 37°C, for 2.0 hours. Reaction was terminated by extraction of RNA with acidic phenol and chloroform followed by ethanol precipitation [17]. Extracted rRNA was adsorbed on DE-81 anion-exchange membrane, washed with 100 mM Glycine-HCl, pH 2.3 followed by ethanol and dried filters were placed in scintillation fluid for radioactive counts in Packard liquid Scintillation Counter, USA. Each reaction was set-up in duplicate.

## Competing interests

The financial assistance for the work was provided by Department of Biotechnology, Government of India, India. None of the authors have any competing parallel interests.

## Authors' contributions

AR had conceived, designed and performed experiments, analyzed the data, wrote manuscript, SSS helped in phagosome preparation, AS helped in screening of Rv1694/pET28a+ construct, NA helped in Ribosome preparation and MVK conceived, designed and analyzed the data and edited the manuscript. All authors read and approved the final manuscript.
